# A multi-center study on the adaptability of a shared foundation model for electronic health records

**DOI:** 10.1038/s41746-024-01166-w

**Published:** 2024-06-27

**Authors:** Lin Lawrence Guo, Jason Fries, Ethan Steinberg, Scott Lanyon Fleming, Keith Morse, Catherine Aftandilian, Jose Posada, Nigam Shah, Lillian Sung

**Affiliations:** 1 Program in Child Health Evaluative Sciences, The Hospital for Sick Children, Toronto, ON Canada; 2 Stanford Center for Biomedical Informatics Research, Stanford University, Palo Alto, CA USA; 3 Division of Pediatric Hospital Medicine, Department of Pediatrics, Stanford University, Palo Alto, CA USA; 4 Division of Hematology/Oncology, Department of Pediatrics, Stanford University, Palo Alto, CA USA; 5 Universidad del Norte, Barranquilla, Colombia; 6 Division of Haematology/Oncology, The Hospital for Sick Children, Toronto, ON Canada

**Keywords:** Computer science, Machine learning

## Abstract

Foundation models are transforming artificial intelligence (AI) in healthcare by providing modular components adaptable for various downstream tasks, making AI development more scalable and cost-effective. Foundation models for structured electronic health records (EHR), trained on coded medical records from millions of patients, demonstrated benefits including increased performance with fewer training labels, and improved robustness to distribution shifts. However, questions remain on the feasibility of sharing these models across hospitals and their performance in local tasks. This multi-center study examined the adaptability of a publicly accessible structured EHR foundation model (FM_SM_), trained on 2.57 M patient records from Stanford Medicine. Experiments used EHR data from The Hospital for Sick Children (SickKids) and Medical Information Mart for Intensive Care (MIMIC-IV). We assessed both adaptability via continued pretraining on local data, and task adaptability compared to baselines of locally training models from scratch, including a local foundation model. Evaluations on 8 clinical prediction tasks showed that adapting the off-the-shelf FM_SM_ matched the performance of gradient boosting machines (GBM) locally trained on all data while providing a 13% improvement in settings with few task-specific training labels. Continued pretraining on local data showed FM_SM_ required fewer than 1% of training examples to match the fully trained GBM’s performance, and was 60 to 90% more sample-efficient than training local foundation models from scratch. Our findings demonstrate that adapting EHR foundation models across hospitals provides improved prediction performance at less cost, underscoring the utility of base foundation models as modular components to streamline the development of healthcare AI.

## Introduction

*Foundation models*^1^, large-scale artificial intelligence (AI) models trained on massive amounts of unlabeled data using self-supervised learning, mark a paradigm shift for healthcare AI by moving away from bespoke, single-purpose models to generalist and more easily adaptable medical AI^2^. Foundation models open new opportunities to improve diagnostic and predictive capabilities, enable proactive interventions and improve patient care using a range of modalities including natural language^3,4^, imaging^5^, genomics^6,7^, and structured data from electronic health records (EHRs)^8–11^. Structured EHR foundation models, trained on tabular, timestamped event data for procedures, diagnoses, medications, and lab values as examples, offer distinct representational abilities over other modalities by focusing on encoding patients’ longitudinal medical history. This enables generating feature representations that summarize a patient’s entire medical history up to a specific time point, facilitating downstream tasks such as risk stratification and time-to-event modeling.

Recent EHR foundation models report state-of-the-art accuracy, require fewer labeled examples for task adaptation, and have demonstrated improved robustness to distribution shifts across time and patient subpopulations^12,13^. With model hubs (centralized repositories for pretrained model weights) playing a key role in modern AI development, sharing EHR foundation models across sites offers many practical advantages by providing a less expensive route for local hospitals to adapt a foundation model for their specific needs. More importantly, key properties of foundation models, such as their skills, domain knowledge, and biases, are highly dependent on the specific data used for pretraining^14,15^. Since large-scale EHR datasets (>1 million patients) are challenging to obtain for most researchers, sharing EHR foundation model weights becomes critical to advancing research into mitigating biases, improving robustness, and other properties intrinsic to a specific set of pretrained model weights. Finally, given recent arguments for regulatory oversight and quality assurance of healthcare AI models by public-private entities^16^, access to foundation model weights that have undergone some certification process may become a prerequisite for model deployment.

Adapting and improving existing foundation models (rather than pretraining from scratch) is the predominant workflow in domains such as NLP and computer vision. However, the absence of public structured EHR foundation models has hampered similar progress in EHR settings^17^. This creates challenges in advancing label/sample efficiency, few-shot learning, and general methods to improving EHR foundation models without access to the original pretraining data^18^. For example, work in other modalities has found that pre-training on large-scale, heterogeneous data generally improves robustness^19^ and that *continued pretraining* of existing models using in-domain data further improves performance in a target domain^20^. This offers a promising route to improving existing EHR foundation models at local hospitals but introduces potential challenges around catastrophic forgetting and other issues that have been underexplored due to the lack of large-scale, shared EHR models.

Although there is a growing body of work evaluating pretrained models across different hospital systems (GenHPF^21^, TransformEHR^22^) and transfer from EHR data to insurance claims (Med-BERT^9^), prior studies have focused on private foundation models, pretrained from scratch, and the role architectural choices play in transfer learning performance in downstream task adaptation. There has been limited exploration of label efficiency in EHR settings, where encoder-only/BERT-style models perform poorly on few-shot tasks. For example, Med-BERT required an average of 200–1000 training examples per adapted task to outperform their reported logistic regression baselines. To our knowledge, no prior work has investigated strategies for improving existing EHR foundation models via continued pretraining or evaluations on how such training impacts label efficiency in downstream, adapted task models.

To address these challenges, we present a multi-center study focused on the adaptability of CLMBR-T-base^23^, a recently released 141 M parameter, decoder-only Transformer model for structured EHR data. This publicly accessible foundation model was pretrained from scratch on longitudinal, structured medical records from 2.57 M patients from Stanford Medicine (FM_SM_) and is compatible with the widely adopted Observational Medical Outcomes Partnership Common Data Model (OMOP CDM). The model’s architecture has undergone extensive evaluation at Stanford Medicine across various settings^8,12,13,23^.

Our main contributions are summarized as follows:

- The first multi-site evaluation of continued pretraining using a structured EHR foundation model. We evaluate cross-site, continued pretraining and adaptation for three foundation models trained from scratch and reflecting three different health systems: Stanford Medicine, SickKids (Toronto), and MIMIC-IV. We find continued pretraining improved performance in all models by an average of 3%.
- We evaluate the impact of continued pretraining on few-shot performance using 8 clinician-curated evaluation tasks and training sizes ranging from 2 to 1024 examples. We find substantial improvements to label efficiency, where on average 128 training examples can match baseline performance of a GBM trained on all available task data. This significantly outperforms prior label efficiency experiments that used encoder-only architectures.
- Continued pretraining results in foundation models that are similar in performance to those trained from scratch, but require 60 to 90% less patient data for pretraining.

## Results

### Cohort characteristics and outcome prevalence

Supplementary Fig. 1 illustrates the assignment of patients into the inpatient cohorts for the two datasets sourced from The Hospital for Sick Children (SickKids) in Toronto, Canada, and Beth Israel Deaconess Medical Center (BIDMC) in Boston, United States. Table 1 presents patient demographic characteristics and prevalence for the 8 clinical prediction tasks within the SickKids and MIMIC inpatient cohorts. The prevalence of outcomes was highly skewed, with MIMIC having higher rates in 6/8 tasks (1.3–6 times larger than SickKids).

**Table 1:** Cohort characteristics and outcome prevalence

	SickKids (*n* = 37,960)	MIMIC (*n* = 44,055)
Median age [IQR]	7 [2, 13]	56 [35, 71]
Sex, *n* (%)		
Male	20,507 (54.0%)	17,329 (39.3%)
Race, *n* (%)		
White	^b^	27,402 (62.2%)
Black or African American	^b^	5338 (12.1%)
Asian	^b^	1799 (4.1%)
Other	^b^	5066 (11.5%)
Unknown or Unable to Obtain	^b^	4450 (10.1%)
^a^Outcomes, *n* (%)		
In-hospital Mortality	216 (0.6%)	1599 (3.6%)
Long LOS	6115 (16.1%)	12,215 (27.7%)
30-day Readmission	2275 (6.0%)	259 (0.6%)
Hypoglycemia	459 (1.2%)	719 (1.6%)
Hyponatremia	92 (0.2%)	385 (0.9%)
Hyperkalemia	352 (0.9%)	375 (0.9%)
Thrombocytopenia	726 (1.9%)	1342 (3.1%)
Anemia	1073 (2.9%)	2954 (6.8%)

*IQR* interquartile range, *LOS* length of stay, *MIMIC* Medical Information Mart for Intensive Care.

^a^Refer to text for outcome definitions.

^b^Race data is not routinely collected at SickKids.

### Overall model performance

**Fig. 1:**
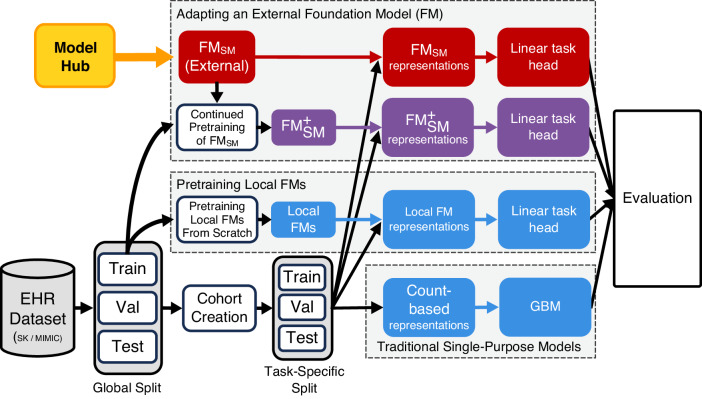
Overview of model training and evaluation. Patients in each dataset (SK and MIMIC) were globally split into training, validation, and test sets. An inpatient cohort was defined for patients in each dataset. We adopted an external FM (FM_SM_), CLMBR-T-base, pretrained on structured EHRs of 2.57 M patients from Stanford Medicine and used it to generate representations for each patient in the cohorts. We also conducted continued pretraining using FM_SM_ on the global training set of each dataset and subsequently constructed patient representations using the resulting models (${{\rm{FM}}}_{{\rm{SM}}}^{+{\rm{SK}}}$ and ${{\rm{FM}}}_{{\rm{SM}}}^{+{\rm{MIMIC}}}$). With these representations, we trained linear task heads (logistic regression) and compared them to locally trained models across the SK and MIMIC datasets and 8 evaluation tasks spanning operational outcomes and anticipated abnormal lab results. Abbreviations: EHR electronic health records, SK SickKids, MIMIC Medical Information Mart for Intensive Care, FM foundation model, FM_SM_ external foundation model Stanford Medicine, CLMBR-T clinical language model based representation – transformer, GBM gradient boosting machines, Val validation.

We evaluated the out-of-the-box performance of the CLMBR-T-base external foundation model (FM_SM_) and its performance with continued pretraining (${\text{FM}}_{\text{SM}}^{+}$). These results were compared against models that were locally trained from scratch, including baseline gradient boosting machines (GBMs) and local foundation models (FMs), as illustrated in Fig. 1.

Table 2 shows comparisons of mean area under the receiver operating characteristic curve (AUROC) and expected calibration error (ECE) for FM_SM_ and ${\text{FM}}_{\text{SM}}^{+}$ vs. the baseline GBMs. Supplementary Table 1 provides comparisons against locally trained FMs. ${\text{FM}}_{\text{SM}}^{+}$ had significantly better AUROC compared to GBM in both SickKids and MIMIC cohorts. ECE was also significantly better for FM_SM_ and ${\text{FM}}_{\text{SM}}^{+}$ vs. GBMs in both cohorts.

**Table 2:** Comparing discrimination and calibration of foundation models vs. baseline GBM approaches at each site^a^

	Discrimination evaluation			Calibration evaluation		
Model	Mean AUROC	Difference [foundation model – GBM]	*P*-value^b^	Mean ECE	Difference [foundation model - GBM]	*P*-value^b^
SickKids						
GBM_SK_	0.855 [0.798, 0.904]			0.015 [0.013, 0.017]		
FM_SM_	0.880 [0.826, 0.928]	0.024 [−0.009, 0.072]	0.184	0.005 [0.003, 0.009]	−0.010 [−0.012, −0.006]	**<0.001**
${\text{FM}}_{\text{SM}}^{+\text{SK}}$	0.901 [0.851, 0.944]	0.044 [0.013, 0.099]	**0.002**	0.006 [0.003, 0.009]	−0.010 [−0.013, −0.006]	**<0.001**
MIMIC						
GBM_MIMIC_	0.807 [0.733, 0.868]			0.016 [0.015, 0.018]		
FM_SM_	0.828 [0.775, 0.875]	0.020 [−0.010, 0.062]	0.230	0.007 [0.004, 0.012]	−0.010 [−0.013, −0.005]	**0.002**
${\text{FM}}_{\text{SM}}^{+\text{MIMIC}}$	0.848 [0.783, 0.895]	0.039 [0.009, 0.075]	**0.006**	0.005 [0.003, 0.009]	−0.011 [−0.013, −0.008]	**<0.001**

*AUROC* area under the receiver operating characteristics curve, *CI* confidence interval, *FM*_*SM*_ external foundation model Stanford Medicine, ${\text{FM}}_{\text{SM}}^{+}$ external foundation model Stanford Medicine with continued pretraining - SK or MIMIC, *SK* SickKids, *MIMIC* Medical Information Mart for Intensive Care, *GBM* gradient boosting machines.

^a^Table shows mean AUROC and ECE across tasks [95% hierarchical bootstrap CI].

^b^Bolded values indicate *P* < 0.05.

**Fig. 2:**
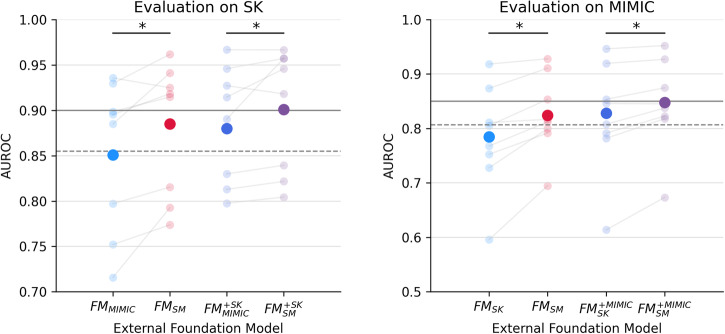
Comparison of external foundation models. Comparing discrimination performance of external foundation models: FMSM vs. FMMIMIC on SK, and FMSM vs. FMSK on MIMIC. Gray lines indicate the performance of locally trained models, with the dashed lines indicating baseline GBMs and solid lines indicating the local foundation models. Bolded and faint dots indicate average and task-specific performance, respectively. Asterisks indicate significant differences at p < 0.05 evaluated using hierarchical bootstrapping. Abbreviations: AUROC area under the receiver operating characteristic curve, FM_SM_/FM_SK_/FM_MIMIC_ external foundation model Stanford Medicine - SK or MIMIC, ${\text{FM}}_{\text{SM}}^{+}$/${\text{FM}}_{\text{SK}}^{+}$/${\text{FM}}_{\text{MIMIC}}^{+}$ external foundation model with continued pretraining – SK or MIMIC, SM Stanford Medicine, SK SickKids, MIMIC Medical Information Mart for Intensive Care.

In an ablation experiment focusing on the adaptability of external foundation models across different hospitals, Fig. 2 demonstrates that FM_SM_ and ${\text{FM}}_{\text{SM}}^{+}$ achieved significantly higher AUROC compared to FM_SK_ and ${\text{FM}}_{\text{SK}}^{+}$ on the MIMIC dataset, and to FM_MIMIC_ and ${\text{FM}}_{\text{MIMIC}}^{+}$ on the SK dataset. See Table 3 for per-task AUROC and Supplementary Table 2 for per-task ECE.

**Table 3:** Discrimination for task-specific models at two sites^a^

Model	In-hospital mortality	Long LOS	30-day readmission	Hypoglycemia	Hyponatremia	Hyperkalemia	Thrombocytopenia	Anemia
Dataset: SickKids								
GBM_SK_	0.893 [0.815, 0.953]	**0.866 [0.853, 0.879]**	0.783 [0.755, 0.809]	0.880 [0.830, 0.924]	0.783 [0.579, 0.957]	0.749 [0.687, 0.808]	0.953 [0.928, 0.975]	0.919 [0.898, 0.938]
FM_MIMIC_	0.885 [0.814, 0.943]	0.797 [0.782, 0.813]	0.752 [0.724, 0.779]	0.899 [0.866, 0.927]	0.936 [0.889, 0.972]	0.716 [0.655, 0.773]	0.930 [0.900, 0.956]	0.895 [0.870, 0.918]
${\text{FM}}_{\text{MIMIC}}^{+\text{SK}}$	0.946 [0.912, 0.973]	0.830 [0.816, 0.844]	0.798 [0.773, 0.821]	**0.927 [0.901, 0.950]**	0.890 [0.789, 0.969]	0.813 [0.767, 0.855]	0.967 [0.953, 0.979]	0.915 [0.892, 0.935]
FM_SM_	0.941 [0.902, 0.971]	0.815 [0.800, 0.830]	0.774 [0.747, 0.799]	0.915 [0.879, 0.945]	0.925 [0.880, 0.963]	0.793 [0.743, 0.838]	0.962 [0.946, 0.975]	0.918 [0.897, 0.937]
${\text{FM}}_{\text{SM}}^{+\text{SK}}$	0.957 [0.922, 0.980]	0.839 [0.825, 0.853]	**0.804 [0.780, 0.828]**	0.918 [0.883, 0.948]	**0.957 [0.933, 0.978]**	0.822 [0.781, 0.860]	**0.967 [0.950, 0.980]**	**0.946 [0.932, 0.959]**
FM_SK_	**0.968 [0.952, 0.981]**	0.847 [0.834, 0.861]	0.774 [0.746, 0.799]	0.924 [0.891, 0.953]	0.936 [0.882, 0.977]	**0.848 [0.812, 0.883]**	0.966 [0.953, 0.978]	0.932 [0.912, 0.950]
Dataset: MIMIC								
GBM_MIMIC_	0.905 [0.886, 0.922]	**0.831 [0.821, 0.841]**	0.619 [0.514, 0.719]	0.790 [0.746, 0.830]	0.798 [0.723, 0.863]	0.700 [0.619, 0.775]	0.915 [0.884, 0.943]	0.878 [0.862, 0.893]
FM_SK_	0.874 [0.852, 0.894]	0.753 [0.741, 0.764]	0.596 [0.49, 0.693]	0.768 [0.728, 0.805]	0.811 [0.762, 0.857]	0.728 [0.657, 0.795]	0.918 [0.896, 0.939]	0.805 [0.786, 0.824]
${\text{FM}}_{\text{SK}}^{+\text{MIMIC}}$	0.920 [0.904, 0.934]	0.792 [0.781, 0.802]	0.614 [0.507, 0.716]	0.808 [0.774, 0.840]	0.846 [0.801, 0.888]	0.782 [0.726, 0.836]	0.946 [0.929, 0.962]	0.854 [0.837, 0.869]
FM_SM_	0.911 [0.894, 0.926]	0.792 [0.781, 0.803]	**0.695 [0.601, 0.781]**	0.812 [0.779, 0.844]	0.817 [0.771, 0.859]	0.800 [0.739, 0.856]	0.928 [0.909, 0.946]	0.854 [0.836, 0.871]
${{\rm{FM}}}_{{\rm{SM}}}^{+{\rm{MIMIC}}}$	0.927 [0.912, 0.941]	0.823 [0.813, 0.833]	0.673 [0.564, 0.771]	0.837 [0.802, 0.869]	**0.845 [0.806, 0.882]**	**0.818 [0.762, 0.868]**	**0.952 [0.939, 0.964]**	0.875 [0.859, 0.890]
FM_MIMIC_	**0.93 [0.916, 0.944]**	0.826 [0.816, 0.836]	0.690 [0.589, 0.784]	**0.838 [0.806, 0.869]**	**0.845 [0.799, 0.887]**	0.814 [0.763, 0.862]	0.950 [0.934, 0.964]	**0.887 [0.872, 0.900]**

*AUROC* area under the receiver operating characteristic curve, *GBM* gradient boosting machines, *FM*_*SM*_*/FM*_*SK*_*/FM*_*MIMIC*_ foundation model Stanford Medicine, SickKids, or MIMIC, ${{\rm{FM}}}_{{\rm{SM}}}^{+}/{{\rm{FM}}}_{{\rm{SK}}}^{+}/{{\rm{FM}}}_{{\rm{MIMIC}}}^{+}$ external foundation model with continued pretraining - SK or MIMIC, *SK* SickKids, *MIMIC* Medical Information Mart for Intensive Care, *LOS* length of stay, *CI* confidence interval.

^a^Table shows AUROC (95% bootstrap CI) for each task; bolded values indicate highest AUROC across models.

### Few-shot performance

**Fig. 3:**
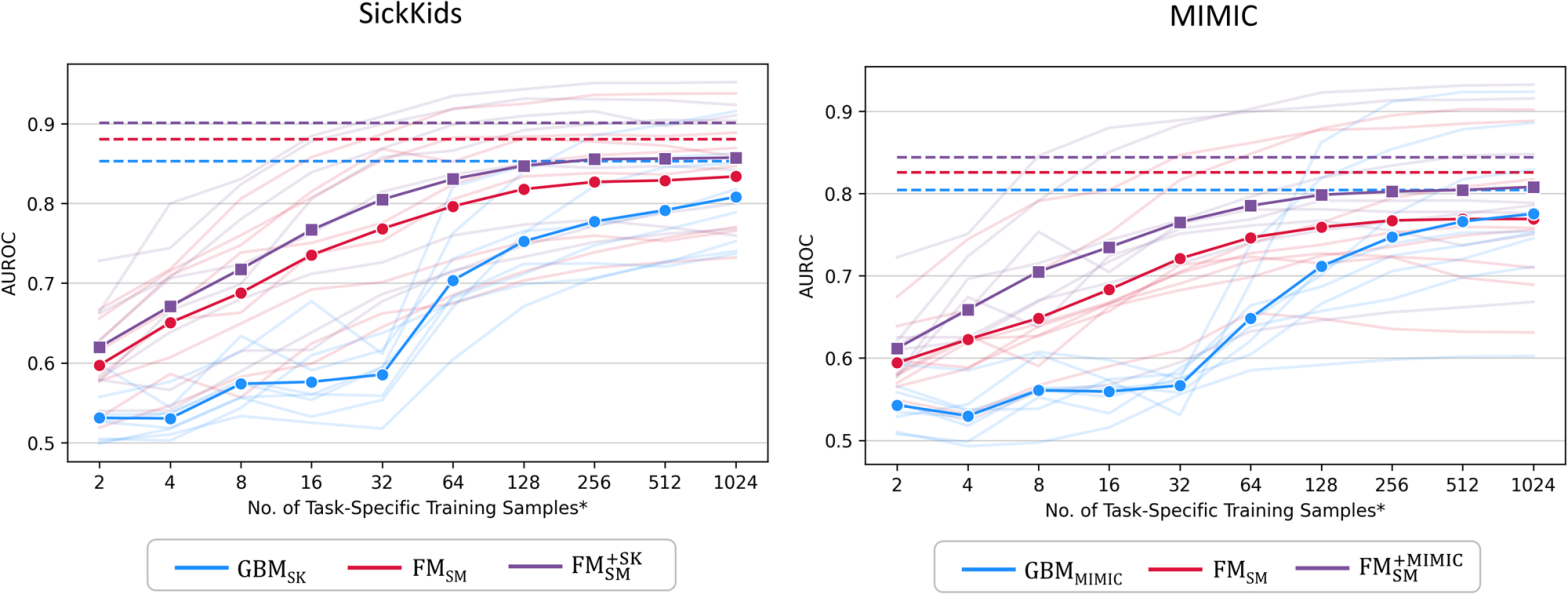
Few-shot performance. Discrimination of external foundation model (FM_SM_), external foundation model with continued pretraining (${\text{FM}}_{\text{SM}}^{+}$) and baseline GBM using decreasing training samples at SickKids and MIMIC. Bolded and faint lines indicate average and task-specific performance, respectively. Dashed lines indicate mean AUROC of models trained on all training samples. * The number of training examples for each class is up to half of the number of task-specific training samples. Abbreviations: AUROC area under the receiver operating characteristic curve, FM_SM_ external foundation model Stanford Medicine, ${\text{FM}}_{\text{SM}}^{+}$ external foundation model Stanford Medicine with continued pretraining – SK or MIMIC, SK SickKids, MIMIC Medical Information Mart for Intensive Care, GBM gradient boosting machines.

In the few-shot experiments, we evaluated the label efficiency of our models by limiting the number of task-specific training examples to values between 2 and 1024, increasing in powers of 2 (see Fig. 3 and Supplementary Table 3). FM_SM_ and ${\text{FM}}_{\text{SM}}^{+}$ consistently demonstrated significantly improved AUROC compared to GBM across almost all few-shot settings in both datasets (13% improvement on average for FM_SM_ and 19% for ${\text{FM}}_{\text{SM}}^{+}$). Furthermore, ${\text{FM}}_{\text{SM}}^{+}$ matched mean AUROC of GBM using as little as 128 samples (64 samples per class; fewer than 1% of all training examples). Supplementary Fig. 2 and Supplementary Table 4 show that FM_SM_ and ${\text{FM}}_{\text{SM}}^{+}$ generally demonstrated lower ECE against GBM although the difference was not statistically significant in some settings, for example in 32-, 512-, and 1024-shot settings for SK.

### Performance with varying pretraining sample size

**Fig. 4:**
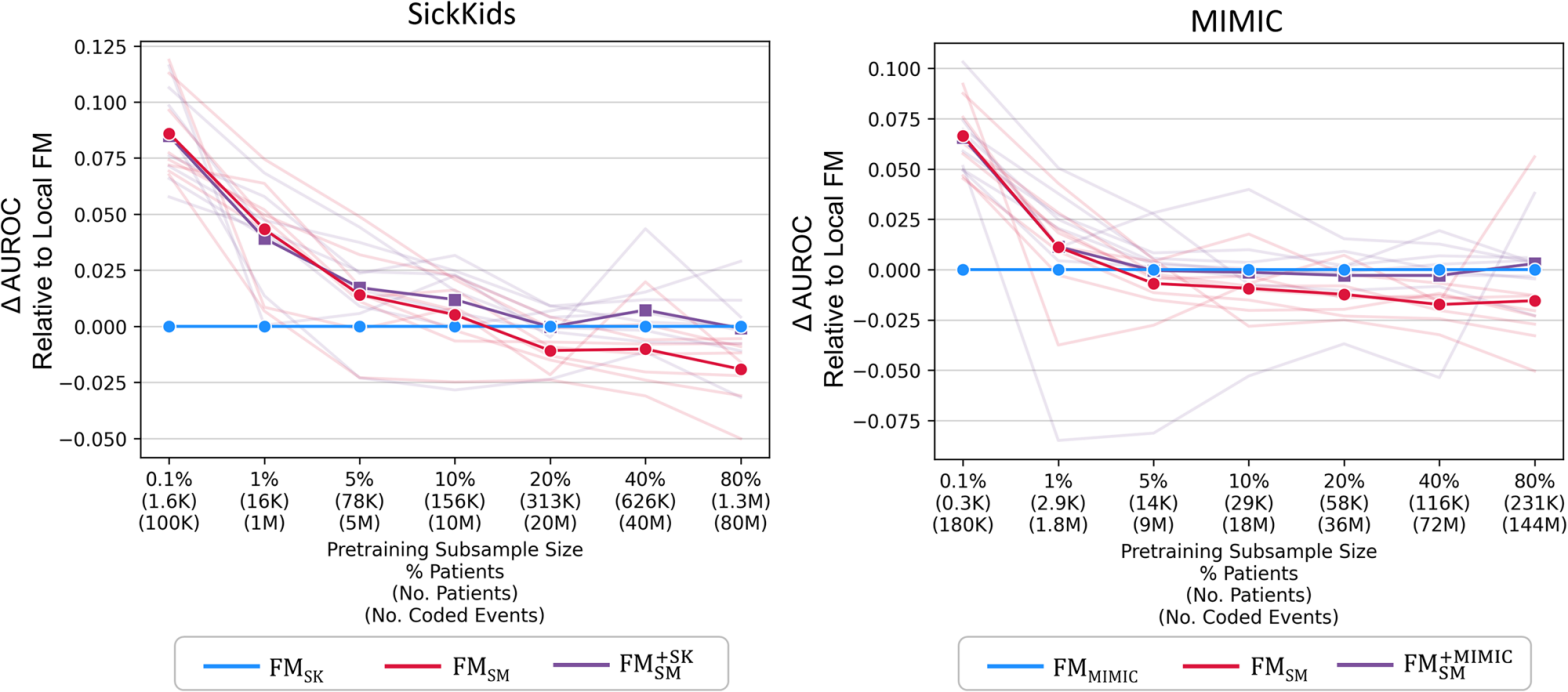
External vs. local foundation models with varying pretraining sample sizes. Discrimination of external foundation model (FM_SM_) and external foundation model with continued pretraining (${\text{FM}}_{\text{SM}}^{+}$) relative to local foundation model (FM_SK_ and FM_MIMIC_) using decreasing pretraining sample size. Bolded and faint lines indicate average and task-specific performance relative to local foundation models, respectively. The subsample size is not relevant for FM_SM_, which did not undergo additional pretraining. Note, the model AUROC scores are relative to the baseline local models (FM_SK_ and FM_MIMIC_) and the absolute AUROC does change across sample sizes. Abbreviations: AUROC area under the receiver operating characteristic curve, FM_SM_ external foundation model Stanford Medicine, ${\text{FM}}_{\text{SM}}^{+}$ external foundation model Stanford Medicine with continued pretraining – SK or MIMIC, FM_SK_/FM_MIMIC_ local foundation models – SK or MIMIC, SK SickKids, MIMIC Medical Information Mart for Intensive Care.

In the experiments examining the impact of varying pretraining sample sizes, we investigated the performance differences between the external foundation models (FM_SM_ and ${\text{FM}}_{\text{SM}}^{+}$) and locally trained foundation models. Figure 4 and Supplementary Table 5 demonstrate that AUROCs for external foundation models were significantly better than local foundation models with very small subsamples (up to 1% for SickKids and 0.1% for MIMIC). Without continued pretraining, AUROC for FM_SK_ was significantly better than FM_SM_ when subsamples reached 80% for SickKids. In contrast, AUROC for local FMs and ${\text{FM}}_{\text{SM}}^{+}$ did not differ significantly across larger subsamples. Calibration results, detailed in Supplementary Fig. 3 and Supplementary Table 6, did not significantly vary between models.

**Fig. 5:**
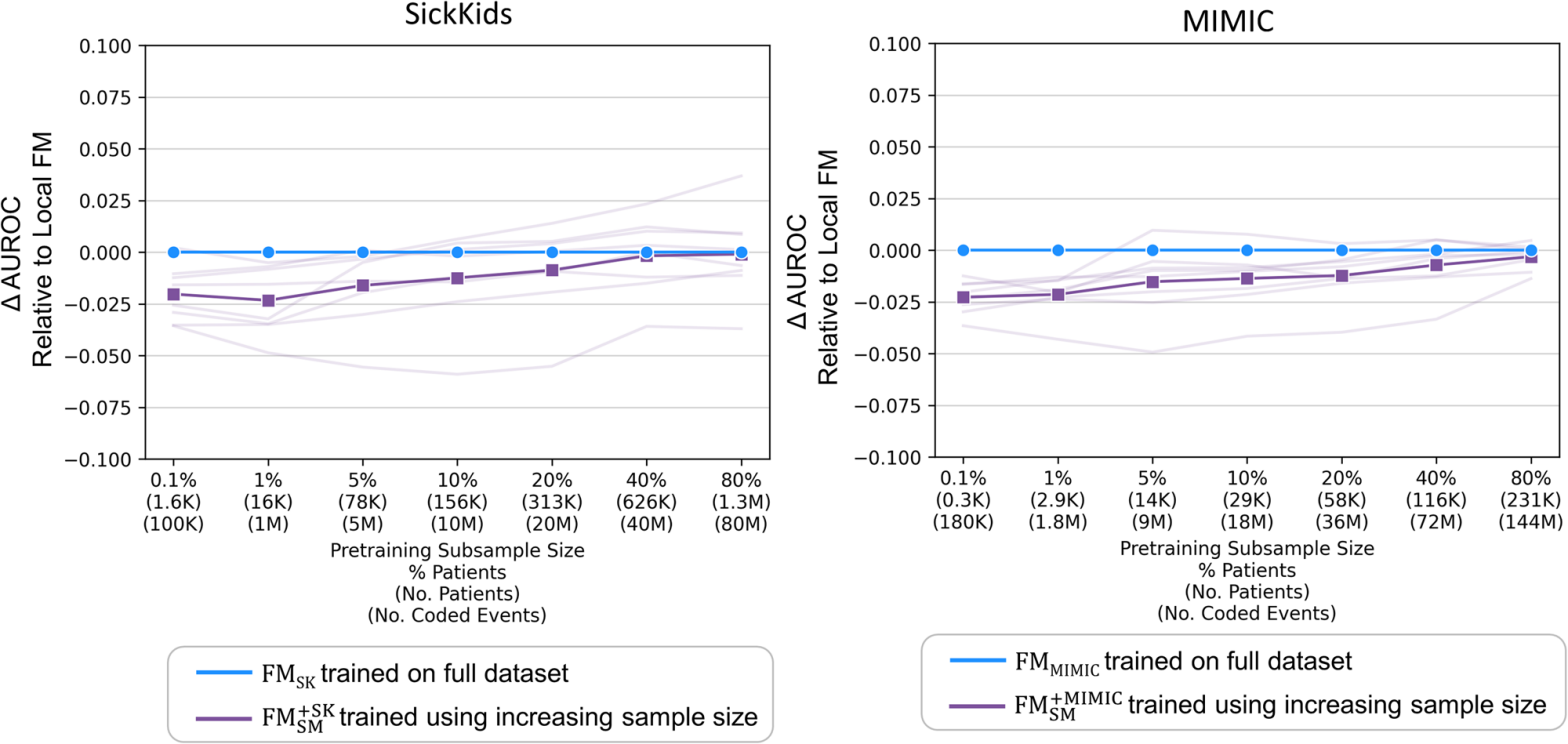
Continued pretraining of external foundation vs. fully trained local foundation model. Discrimination of ${\text{FM}}_{\text{SM}}^{+}$ (external foundation model Stanford Medicine with continued pretraining) using increasing pretraining sample size relative to discrimination of local foundation models (FM_SK_ and FM_MIMIC_) pretrained using all data. Bolded and faint lines indicate average and task-specific performance relative to local foundation models (FM_SK_ and FM_MIMIC_), respectively. Abbreviations: AUROC area under the receiver operating characteristic curve, ${\text{FM}}_{\text{SM}}^{+}$ external foundation model Stanford Medicine with continued pretraining - SK or MIMIC, FM_SK_/FM_MIMIC_ local foundation model – SK or MIMIC, SK SickKids, MIMIC Medical Information Mart for Intensive Care.

Figure 5 and Supplementary Table 7 show that the performance of continued pretraining with subsamples of 10% or more for SK and 40% or more for MIMIC did not significantly differ from that of local foundation models (FM_SK_ and FM_MIMIC_) trained from scratch on all data. In addition, continued pretraining was more efficient than pretraining from scratch (70.2% faster than FM_SK_ and 58.4% faster than FM_MIMIC_), as shown in Supplementary Fig. 4. Furthermore, as demonstrated in Supplementary Fig. 5, FM_SM_ and ${\text{FM}}_{\text{SM}}^{+}$ generally processed fewer coded events (50.9% less in SK and 30.9% less in MIMIC) than local foundation models as a result of reduced code coverage.

## Discussion

Our multi-center study has demonstrated that adapting an off-the-shelf external foundation model (FM_SM_) can yield comparable discrimination and better calibration compared to baseline GBM models locally trained on all available data at each site, while providing 13% discrimination improvement in settings with few task-specific training labels. With continued pretraining on local data, significantly better discrimination and calibration were observed compared to baseline GBM models. In addition, label efficiency substantially improved, such that using only 128 training examples (64 samples per class; less than 1% of the total data), ${\text{FM}}_{\text{SM}}^{+}$ matched the performance of the GBMs training using all available data. Furthermore, continued pretraining of FM_SM_ requires 60 to 90% less patient data than local pretraining from scratch to achieve the same level of performance, demonstrating better sample efficiency. Our findings also provided insights into when it is beneficial to adapt an existing EHR foundation model vs. pretraining from scratch, depending on data availability.

The development of single-purpose models entails significant costs^24^. These costs escalate further for EHR foundation models, which require access to extensive patient records and substantial computing resources. Our findings highlight the potential for cost savings by sharing and building upon pretrained EHR foundation models across hospitals. Adapting these models to new tasks significantly reduces the amount of training labels needed, thereby lowering label acquisition costs and speeding up the deployment of new applications. Moreover, for institutions equipped to conduct pretraining, the continued pretraining of an existing foundation model is considerably more data- and compute-efficient compared to pretraining from scratch.

Our results contribute to recent work demonstrating the robustness of EHR foundation models to various distribution shifts^12,13^. Compared to traditional single-purpose models that often fall short in adaptability across sites^25,26^, EHR foundation models demonstrate a notable capacity to encode patients’ longitudinal medical history and local care nuances. Despite challenges posed by dataset shifts, notably in patient populations and coding variations as evidenced by the number of unprocessed codes by FM_SM_ across all patient timelines at each site, the external foundation model displayed robust performance. Moreover, our results indicate that pretraining on a larger and more diverse patient population improves the adaptability of the foundation model across healthcare settings. It is noteworthy that a single external foundation model consistently achieved strong performance across both a Canadian pediatric cohort and an American adult ICU-based cohort. Our findings point towards a paradigm where, instead of training bespoke models for each healthcare site from scratch, the focus shifts to the development and sharing of larger, general-purpose base foundation models and recipes for site-specific refinement, such as continued pretraining.

To leverage the transfer learning capabilities of pretrained foundation models, it is important to adhere to the underlying data schema and vocabulary of tokens used by these models. For this reason, we mapped our datasets to the OMOP CDM. Progress is required to define minimal schema requirements for training foundation models to mitigate costs associated with mapping to a common data model. Alternatively, developing EHR foundation models that are robust to schema shifts^27^, akin to multilingual language models, represents a valuable direction for future research.

Sharing foundation models across hospitals needs to address privacy and ethical use of the underlying data. For EHR foundation models, adopting measures such as training on data de-identified to HIPAA standards, acquiring patient consent, and restricting access to accredited individuals^11^ are important first steps. Despite these efforts, issues like potential misuse (e.g., for surveillance) and the challenge of algorithmic biases remain open research questions^1,28^, underscoring the ongoing challenges in maintaining privacy and mitigating risks in medical foundation models.

This study has several strengths and limitations. A primary strength of this study is that it represents one of the first external evaluation of a publicly available foundation model specifically for structured EHR data. Additionally, we have characterized the utility of the foundation model across diverse evaluation settings. On the limitation side, this study was conducted using a limited number of hospital datasets and tasks, which may not capture the full spectrum of EHR variability. We did not explore methods for harmonizing across schemas, which could impact the adaptability of the foundation model. We do not explore trade-offs of relative set sizes of training vs validation data when conducting few-shot learning, which may impact reported performance. We also did not explore questions on fairness and the propagation of biases potentially associated with sharing foundation models. Moreover, the study emphasized a specific foundation model, CLMBR-T-base, due to lack of publicly available structured EHR foundation models^17^. Lastly, while the benefits of continued pretraining are clear, they may not be accessible to institutions that lack resources to perform it, potentially limiting broader applicability.

In conclusion, our findings show that adapting a pretrained EHR foundation model for downstream tasks across hospitals can improve prediction performance at less cost, underscoring the effectiveness of sharing base foundation models as modular machine learning components to streamline the development of healthcare AI.

## Methods

### Hospital datasets

This retrospective multi-center study utilized EHR data collected from two institutions, The Hospital for Sick Children (SickKids) in Toronto, Canada, and Beth Israel Deaconess Medical Center (BIDMC) in Boston, United States.

The SickKids (SK) dataset was sourced from the SickKids Enterprise-wide Data in Azure Repository (SEDAR)^29^ and contains EHR data for 1.8 M patients collected from June 2018 to March 2023. The MIMIC dataset (MIMIC-IV version 1.0)^30,31^ encompasses data from 340 K patients admitted to an intensive care unit (ICU) or the emergency department at BIDMC between 2008 and 2019. To meet the schema requirements of the external foundation model, both datasets are mapped to the widely adopted Observational Medical Outcomes Partnership Common Data Model (OMOP-CDM). Mapping for MIMIC was done using code from the MIMIC project as part of Observational Health Data Sciences and Informatics^32^.

The SickKids Research Ethics Board (REB) approved the use of SEDAR for this research (REB number: 1000074527). Data from MIMIC-IV was approved under the oversight of the Institutional Review Boards (IRB) of BIDMC and Massachusetts Institute of Technology (MIT). Data access was credentialed under the oversight of the data use agreement through PhysioNet^31^.

### Models

Our baseline approach employed a conventional count-based featurization approach^33,34^ to create representations for each patient, task, and cohort, detailed further in Supplementary Table 8. Using these representations, we trained GBM models using LightGBM^35^ on the task-specific training sets, resulting in GBM_SK_ for SK and GBM_MIMIC_ for MIMIC. We considered the choice of optimization algorithm as a hyperparameter. Detailed hyperparameter settings are available in Supplementary Table 9, with tuning performed on the task-specific validation sets to optimize for log loss.

For the external foundation model (FM_SM_), we selected CLMBR-T-base^23^ because it is the only publicly available EHR foundation model that has also undergone extensive evaluation^8,12,13,23^. FM_SM_ processes sequences of clinical events as inputs, where each sequence $X=({x}_{1},{x}_{2},\,\ldots ,\,{x}_{n})$ represents the medical timeline of an individual patient, with each ${x}_{i}$ denoting the *i*-th code, encompassing any form of structured data obtained from the patient’s EHR, such as a diagnosis, procedure, medication, or lab test as examples. The architecture of the model comprises 12 stacked transformer layers with a local attention mechanism, totaling 141 million parameters. It was pretrained on structured EHRs from 2.57 million patients receiving care at Stanford Health Care and Lucile Packard Children’s Hospital between 2008 and 2022. CLMBR-T-base is a decoder-only model, autoregressively pretrained to predict the next clinical event ${x}_{t+1}$ based on the preceding sequence, similar to GPT pretraining. The vocabulary is defined as the top 65,536 codes from the union of all codes in 21 source ontology mappings (e.g. LOINC, SNOMED, RxNorm) provided by Athena’s OMOP vocabulary list. The top codes were selected based on global code frequency in the original pretraining dataset. Codes not in the vocabulary were dropped.

In our experiments, we used FM_SM_ out-of-the-box as a frozen feature encoder, transforming the sequence of clinical events *X* for each patient into a dense vector representation $R={f}_{\theta }(X)$, with the model’s parameters *θ* fixed during this encoding process. The obtained representations were then used to train linear task heads (also known as linear probes^36^) on the task-specific training sets. Specifically, we utilized L2-regularized logistic regression models from Sci-kit Learn^37^ with the LBFGS solver as our linear task heads. We conducted hyperparameter tuning on the task-specific validation sets (see Supplementary Table 2 for hyperparameter settings) to optimize for log loss.

In addition, we tailored FM_SM_ to each dataset separately by performing continued pretraining, using patient timelines from each dataset, resulting in ${\text{FM}}_{\text{SM}}^{+\text{SK}}$ and ${\text{FM}}_{\text{SM}}^{+\text{MIMIC}}$. Continued pretraining uses the same next-code, autoregressive prediction task used during the original pretraining run. Pretraining resumed on the global training set, tuning the learning rate using global validation set performance. Pretraining continued for up to 10 million steps, with early stopping implemented if the validation loss did not improve for 15,000 steps. Once continued pretraining was complete, the training of linear task heads followed the same procedure as FM_SM._

Lastly, we pretrained local foundation models from scratch on each dataset using up to 1.6 M patients (100 M coded events) for SK and 290 K (180 M coded events) for MIMIC, resulting in FM_SK_ and FM_MIMIC_. FM_SK_ and FM_MIMIC_ shared the same architecture as FM_SM_. Hyperparameter tuning followed the same procedure as continued pretraining_._

### Computation resources

Pretraining was executed on an on-premises cluster of up to 4 Nvidia V100 GPUs.

### Clinical prediction tasks, prediction times, and observation window

We defined 8 binary clinical prediction tasks. The binary classification setting is standard and particularly well-suited for the class of models being evaluated, and the tasks were selected based on clinical relevance, alignment with previous benchmarks^13,23^, and previous validation work^38^. The tasks were categorized into two groups: operational tasks and predicting clinically relevant abnormal lab results. Operational tasks encompassed in-hospital mortality, long length of stay of at least 7 days (long LOS), readmission within 30 days of discharge (30-day readmission). Tasks related to predicting abnormal lab-based results included hypoglycemia (serum glucose <3 mmol/L)^39^, hyponatremia (serum sodium <125 mmol/L)^40^, hyperkalemia (serum potassium >7 mmol/L)^41^, anemia (hemoglobin <70 g/L)^42^, and thrombocytopenia (platelet count <50 ×10^9^/L).

The prediction time for the 30-day readmission task was set at midnight on the day of discharge. For all other tasks, the prediction time was set at midnight on the day of admission. The prediction window extended until discharge for all tasks, with the exception of the readmission task, which utilized a 30-day window post-discharge. The observation window for model input encompassed all available patient data up to the prediction time.

### Inpatient cohort and data splitting procedure

SickKids inpatients were those 28 days of age or older at admission. MIMIC inpatients were those 18 years of age or older at admission. In cases where patients had multiple admissions, we randomly selected one admission for inclusion. We excluded admissions in which patient death or discharge occurred between admission and prediction time. Additionally, for each clinical prediction task, we excluded admissions in which the outcome occurred between admission (or discharge) and prediction time.

We defined global training, validation, and test sets for each dataset in a 70/15/15 ratio across all patients used for continued pretraining and local foundation models. Subsequently, for each inpatient cohort, we derived task-specific training, validation, and test sets. The task-specific sets were subsets of the global sets, thereby preserving the 70/15/15 distribution.

### Experiments

We assessed models’ overall performance by comparing discrimination (AUROC) and calibration (expected calibration error, ECE) of FM_SM_ and FM_SM_ with continued pretraining (${\text{FM}}_{\text{SM}}^{+})$ vs. the baseline GBM across all clinical prediction tasks using the task-specific test set of each dataset. We also compared discrimination and calibration of FM_SM_ and ${\text{FM}}_{\text{SM}}^{+}$ with local foundation models (FM_SK_ and FM_MIMIC_). In an ablation experiment, we evaluated the adaptability of FM_SM_ against FM_SK_ and FM_MIMIC_, hypothesizing that FM_SM_’s training on a larger and more heterogenous patient population makes it more adaptable across healthcare sites. To this end, we examined the performance of FM_SK_ on MIMIC and FM_MIMIC_ on SK as external foundation models, including their performance with continued pretraining (${\text{FM}}_{\text{SK}}^{+\text{MIMIC}}$ and ${\text{FM}}_{\text{MIMIC}}^{+\text{SK}}$), compared to FM_SM_ and ${\text{FM}}_{\text{SM}}^{+}$.

Next, we compared FM_SM_ and continued pretraining of FM_SM_ ${(\text{FM}}_{\text{SM}}^{+})$ against the baseline GBM in the setting of decreasing training samples to evaluate label efficiency. For each task, we varied the number of training examples (*k)* within (2, 4, 8, 16, 32, 64, 128, 256, 512, 1024). These examples consisted of an equal number of positive and negative instances, except for tasks with *k*_*p*_ positive instances, where *k*_*p*_ < *k/2*. For such tasks, we included all *k*_*p*_ positive instances in our training data. The training examples were drawn from a subset of the task-specific training set, while validation and test sets remained the same. We do not k-sample the validation set under the assumption that hospitals, in practice, will have access to larger task sets for evaluating model performance. In order to ensure an unbiased performance estimate, we removed all task-specific training sets from pretraining for these experiments to ensure that pretraining is not unfairly advantaged by being able to see more examples of a particular task. The procedures for training baseline GBM and linear task heads for foundation models were kept consistent for the few-shot experiments. We performed 20 iterations of sampling, task-specific training, and evaluation for each value of *k*. Notably, our method of training linear task heads on FM_SM_ and ${\text{FM}}_{\text{SM}}^{+}$ across all experiments does not involve fine-tuning the foundation model parameters, representing a conservative approach. In contrast, the baseline GBMs underwent extensive hyperparameter tuning, including adjustments to the optimization algorithm, to optimize performance.

Lastly, we evaluated the effect of decreasing pretraining sample size on the performance of continued pretraining (${{\rm{FM}}}_{{\rm{SM}}}^{+{\rm{SK}}}$ and ${{\rm{FM}}}_{{\rm{SM}}}^{+{\rm{MIMIC}}}$) and pretraining local foundation models from scratch (FM_SK_ and FM_MIMIC_). Our aims were twofold: 1) to provide insight into the decision of whether to employ or tailor (via continued pretraining) FM_SM_ versus pretraining from scratch, depending on sample size availability; and 2) to determine if continued pretraining is more sample efficient than pretraining from scratch. For the first aim, we compared the performance of FM_SM_ with and without continued pretraining against local FMs at each sample size. For the second aim, we assessed how continued pretraining at various sample sizes compared to local FMs trained on the entire dataset. The subsamples used varied from 0.1% to 80% of the global training set, for both continued pretraining and pretraining from scratch.

### Model evaluation and statistical analysis

We evaluated each model’s discrimination performance in the task-specific test sets using the AUROC. Calibration was measured in terms of the ECE using 10 quantile bins for the predicted risks. To calculate ECE in the few-shots experiment, we determined the corrected predicted risk *p’* based on the predicted risk *p* and the task-specific outcome rate *b’* using Formula (1). The correction accounts for biases in the model’s predictions as a result of balanced sampling used during few-shot training^43^.

1 $${p}^{{\prime} }=\frac{p}{p+(1-p)(1-{b}^{{\prime} })/b{\prime} }$$

Differences between model performances were statistically evaluated using a hierarchical bootstrapping method, which samples both patients and outcomes with replacement^44^. In the few-shots experiments, we averaged the performance across all sampling iterations. For each statistical evaluation, we calculated a two-tailed *P*-value by multiplying the one-sided *P*-value by 2, where the one-sided *P-*value represents the smaller proportion of bootstrap differences either to the left of 0 (the null value) or to the right of 0^45^. For all tests, a *P*-value of <0.05 was considered statistically significant.

### Reporting summary

Further information on research design is available in the Nature Research Reporting Summary linked to this article.

### Supplementary information

Supplementary Information

Reporting Summary

## Data Availability

The SickKids dataset cannot be made publicly available because of the potential risk to patient privacy. However, relevant data are available upon reasonable request. The MIMIC-IV dataset is available from https://mimic.mit.edu/docs/iv/. Details of the CLMBR-T-base external foundation model are available from https://ehrshot.stanford.edu/. Full model weights of CLMBR-T-base are available from https://huggingface.co/StanfordShahLab/clmbr-t-base.

## Code availability

The code for the analyses conducted in this study is open-source and available at https://github.com/sungresearch/femr-on-sk and https://github.com/sungresearch/femr-on-mimic.
