# Supplementary material for: A multi-center study on the adaptability of a shared foundation model for electronic health records
Source: NPJ Digit Med. 2024 Jun 27;7:171. doi: 10.1038/s41746-024-01166-w (PMC11211479; doi:10.1038/s41746-024-01166-w)
Supplement: Supplementary file 1 — Supplementary Information [file 41746_2024_1166_MOESM1_ESM.pdf]

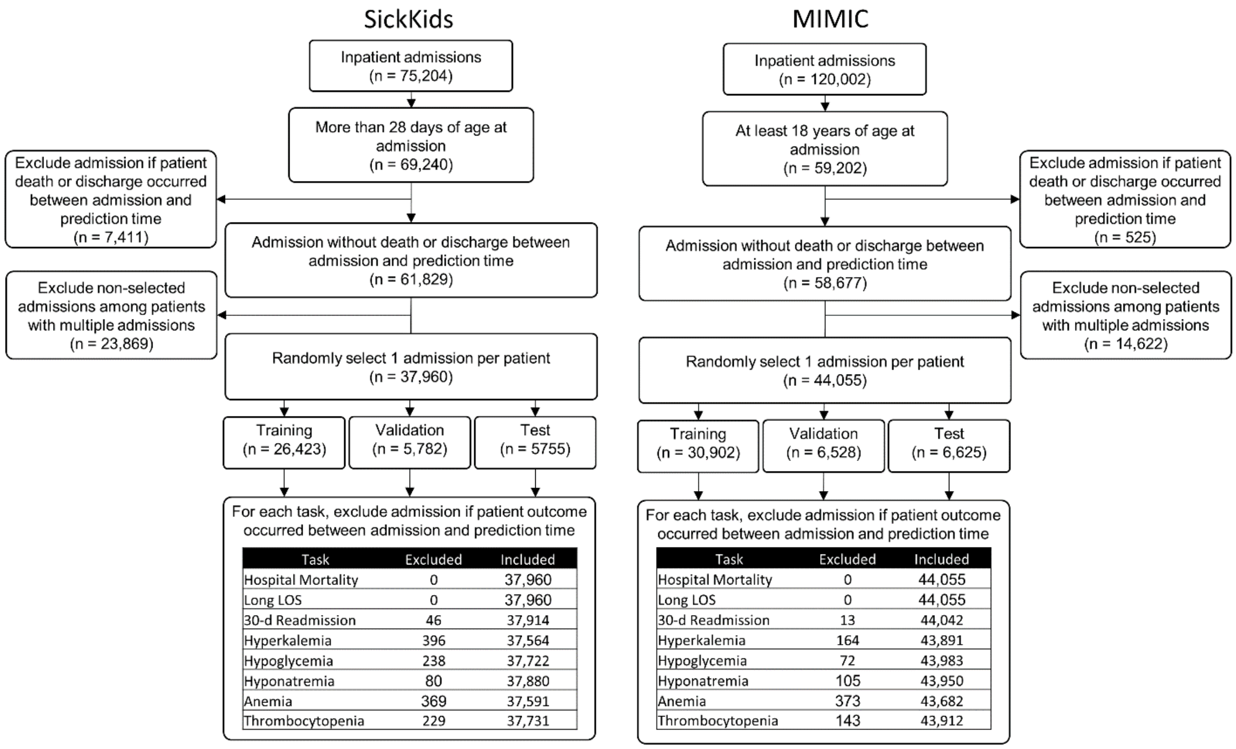

SickKids

Task	Excluded	Included
Hospital Mortality	0	37,960
Long LOS	0	37,960
30-d Readmission	46	37,914
Hyperkalemia	396	37,564
Hypoglycemia	238	37,722
Hyponatremia	80	37,880
Anemia	369	37,591
Thrombocytopenia	229	37,731

MIMIC

Task	Excluded	Included
Hospital Mortality	0	44,055
Long LOS	0	44,055
30-d Readmission	13	44,042
Hyperkalemia	164	43,891
Hypoglycemia	72	43,983
Hyponatremia	105	43,950
Anemia	373	43,682
Thrombocytopenia	143	43,912

Supplementary Figure 1. Flow diagram of patient selection, inclusion, reason for exclusion and assignment into training, validation, and test sets for task-specific models for each dataset. Patients in the test set were excluded from all pretraining of foundation models.
Abbreviation: LOS: length of stay

Supplementary Table 1. Comparing discrimination and calibration of external vs. local foundation models at each site*

	Discrimination Evaluation			Calibration Evaluation		
Model	Mean AUROC	Difference [External FM – Local FM]	P-value**	Mean ECE	Difference [External FM – Local FM]	P-value**
**SickKids**						
FM_{SK}	0.900 [0.849, 0.942]			0.006 [0.003, 0.009]		
FM_{SM}	0.880 [0.826, 0.928]	-0.019 [-0.035, -0.005]	**0.008**	0.005 [0.003, 0.009]	0 [-0.002, 0.002]	0.816
FM_{SM}^{+SK}	0.901 [0.851, 0.944]	0.002 [-0.012, 0.017]	0.774	0.006 [0.003, 0.009]	0 [-0.001, 0.002]	0.848
**MIMIC**						
FM_{MIMIC}	0.850 [0.793, 0.898]			0.006 [0.004, 0.01]		
FM_{SM}	0.828 [0.775, 0.875]	-0.023 [-0.035, -0.006]	**0.014**	0.007 [0.004, 0.012]	0 [-0.001, 0.004]	0.761
FM_{SM}^{+MIMIC}	0.848 [0.783, 0.895]	-0.003 [-0.016, 0.007]	0.522	0.005 [0.003, 0.009]	-0.001 [-0.003, 0.001]	0.326

* Table shows mean AUROC and ECE across tasks [95% hierarchical bootstrap CI]

** Bolded values indicate $P < 0.05$

Abbreviations: AUROC: area under the receiver operating characteristics curve; ECE: expected calibration error; CI: confidence interval; FM_{SM}: external foundation model Stanford Medicine; FM_{SM}^{+}: external foundation model Stanford Medicine with continued pretraining - SK or MIMIC; FM_{SK} or FM_{MIMIC}: local foundation model – SK or MIMIC; SK: SickKids; MIMIC: Medical Information Mart for Intensive Care.

Supplementary Table 2. Calibration for task-specific models at two sites*

	In-hospital Mortality	Long LOS	30-day Readmission	Hypoglycemia	Hyponatremia	Hyperkalemia	Thrombocytopenia	Anemia
Dataset: SickKids								
GBM_{SK}	0.013 [0.012, 0.015]	0.015 [0.011, 0.025]	0.014 [0.010, 0.02]	0.015 [0.013, 0.018]	0.013 [0.012, 0.015]	0.011 [0.009, 0.014]	0.018 [0.016, 0.020]	0.017 [0.015, 0.021]
FM_{MIMIC}	0.002 [0.001, 0.005]	0.011 [0.008, 0.021]	0.007 [0.006, 0.014]	**0.003 [0.002, 0.007]**	0.002 [0.001, 0.003]	**0.005 [0.004, 0.009]**	0.002 [0.002, 0.006]	0.004 [0.003, 0.008]
FM_{MIMIC}^{+SK}	**0.001 [0.001, 0.003]**	0.007 [0.007, 0.019]	0.009 [0.007, 0.016]	0.004 [0.002, 0.007]	**0.001 [0.001, 0.002]**	**0.005 [0.003, 0.009]**	0.002 [0.001, 0.005]	0.005 [0.003, 0.009]
FM_{SM}	0.002 [0.001, 0.004]	0.011 [0.009, 0.022]	**0.004 [0.004, 0.012]**	0.004 [0.002, 0.007]	**0.001 [0.001, 0.002]**	**0.005 [0.003, 0.008]**	**0.001 [0.001, 0.005]**	**0.003 [0.003, 0.008]**
FM_{SM}^{+SK}	0.002 [0.001, 0.004]	0.01 [0.008, 0.021]	0.007 [0.006, 0.014]	0.004 [0.002, 0.007]	**0.001 [0.001, 0.002]**	**0.005 [0.003, 0.008]**	**0.001 [0.001, 0.005]**	0.005 [0.004, 0.009]
FM_{SK}	0.002 [0.001, 0.004]	**0.006 [0.006, 0.017]**	0.006 [0.006, 0.015]	0.005 [0.003, 0.008]	**0.001 [0.001, 0.002]**	0.006 [0.003, 0.009]	0.002 [0.001, 0.005]	0.005 [0.004, 0.01]
Dataset: MIMIC								
GBM_{MIMIC}	0.014 [0.013, 0.019]	0.017 [0.011, 0.027]	0.015 [0.013, 0.016]	0.014 [0.011, 0.017]	0.017 [0.014, 0.019]	0.017 [0.015, 0.019]	0.019 [0.016, 0.022]	0.015 [0.011, 0.021]
FM_{SK}	0.004 [0.003, 0.009]	0.029 [0.023, 0.040]	0.004 [0.003, 0.007]	0.003 [0.003, 0.007]	0.003 [0.002, 0.005]	0.002 [0.002, 0.005]	**0.002 [0.002, 0.007]**	0.008 [0.006, 0.016]
FM_{SK}^{+MIMIC}	0.004 [0.002, 0.009]	0.022 [0.015, 0.032]	**0.002 [0.002, 0.005]**	0.004 [0.003, 0.008]	**0.002 [0.002, 0.004]**	0.003 [0.002, 0.006]	0.004 [0.003, 0.007]	**0.004 [0.004, 0.011]**
FM_{SM}	0.003 [0.003, 0.008]	0.02 [0.016, 0.032]	**0.002 [0.002, 0.004]**	0.004 [0.003, 0.007]	**0.002 [0.002, 0.005]**	0.003 [0.002, 0.005]	**0.002 [0.002, 0.007]**	0.007 [0.006, 0.014]
FM_{SM}^{+MIMIC}	**0.002 [0.002, 0.007]**	**0.009 [0.008, 0.022]**	**0.002 [0.002, 0.005]**	0.003 [0.002, 0.006]	0.003 [0.002, 0.005]	**0.002 [0.002, 0.005]**	0.003 [0.003, 0.007]	0.005 [0.004, 0.012]
FM_{MIMIC}	0.006 [0.003, 0.010]	0.011 [0.009, 0.023]	**0.002 [0.002, 0.005]**	**0.002 [0.002, 0.006]**	0.003 [0.002, 0.005]	**0.002 [0.002, 0.004]**	0.004 [0.003, 0.007]	0.008 [0.006, 0.015]

*Table shows ECE (95% bootstrap CI) for each task; bolded values indicate lowest ECE across models

Abbreviations: ECE: expected calibration error; GBM: gradient boosting machines; FM_{SM}/ FM_{SK}/FM_{MIMIC}: foundation model Stanford Medicine, SickKids, or MIMIC; FM_{SM}^{+}/FM_{SK}^{+}/FM_{MIMIC}^{+}: external foundation model with continued pretraining - SK or MIMIC; SK: SickKids; MIMIC: Medical Information Mart for Intensive Care; LOS: length of stay; CI: confidence interval

Supplementary Table 3. Comparing discrimination of foundation models vs. baseline GBM using decreasing training samples*

Training Samples	Foundation Model	Foundation Model Performance	GBM Performance (Baseline)	Difference [Foundation Model – GBM]	P-value **
Dataset: SickKids					
2	FM_{SM}	0.598 [0.561, 0.631]	0.53 [0.512, 0.555]	0.066 [0.037, 0.096]	**< 0.001**
4	FM_{SM}	0.652 [0.606, 0.689]	0.53 [0.517, 0.546]	0.121 [0.084, 0.151]	**< 0.001**
8	FM_{SM}	0.691 [0.627, 0.742]	0.573 [0.553, 0.598]	0.114 [0.067, 0.160]	**< 0.001**
16	FM_{SM}	0.738 [0.674, 0.793]	0.575 [0.547, 0.61]	0.161 [0.097, 0.216]	**< 0.001**
32	FM_{SM}	0.77 [0.711, 0.830]	0.586 [0.558, 0.609]	0.185 [0.127, 0.239]	**< 0.001**
64	FM_{SM}	0.798 [0.737, 0.854]	0.704 [0.666, 0.748]	0.093 [0.055, 0.127]	**< 0.001**
128	FM_{SM}	0.82 [0.760, 0.870]	0.752 [0.713, 0.795]	0.066 [0.039, 0.096]	**< 0.001**
256	FM_{SM}	0.829 [0.773, 0.876]	0.777 [0.735, 0.820]	0.049 [0.032, 0.074]	**< 0.001**
512	FM_{SM}	0.83 [0.779, 0.876]	0.792 [0.749, 0.835]	0.037 [0.019, 0.057]	**< 0.001**
1024	FM_{SM}	0.835 [0.787, 0.878]	0.809 [0.768, 0.850]	0.025 [0.012, 0.040]	**< 0.001**
2	FM_{SM}^{+SK}	0.621 [0.581, 0.661]	0.53 [0.512, 0.555]	0.089 [0.061, 0.120]	**< 0.001**
4	FM_{SM}^{+SK}	0.674 [0.614, 0.726]	0.53 [0.517, 0.546]	0.142 [0.092, 0.190]	**< 0.001**
8	FM_{SM}^{+SK}	0.719 [0.660, 0.776]	0.573 [0.553, 0.598]	0.144 [0.101, 0.190]	**< 0.001**
16	FM_{SM}^{+SK}	0.77 [0.698, 0.830]	0.575 [0.547, 0.610]	0.193 [0.122, 0.253]	**< 0.001**
32	FM_{SM}^{+SK}	0.807 [0.745, 0.865]	0.586 [0.558, 0.609]	0.222 [0.161, 0.275]	**< 0.001**
64	FM_{SM}^{+SK}	0.833 [0.771, 0.885]	0.704 [0.666, 0.748]	0.127 [0.091, 0.160]	**< 0.001**
128	FM_{SM}^{+SK}	0.85 [0.791, 0.899]	0.752 [0.713, 0.795]	0.095 [0.068, 0.123]	**< 0.001**
256	FM_{SM}^{+SK}	0.858 [0.804, 0.904]	0.777 [0.735, 0.820]	0.078 [0.061, 0.100]	**< 0.001**
512	FM_{SM}^{+SK}	0.858 [0.807, 0.902]	0.792 [0.749, 0.835]	0.064 [0.05, 0.081]	**< 0.001**
1024	FM_{SM}^{+SK}	0.86 [0.809, 0.904]	0.809 [0.768, 0.850]	0.049 [0.036, 0.065]	**< 0.001**
Dataset: MIMIC					
2	FM_{SM}	0.593 [0.569, 0.626]	0.543 [0.524, 0.562]	0.051 [0.033, 0.076]	**< 0.001**
4	FM_{SM}	0.621 [0.583, 0.664]	0.529 [0.510, 0.550]	0.092 [0.068, 0.118]	**< 0.001**
8	FM_{SM}	0.651 [0.611, 0.700]	0.561 [0.538, 0.583]	0.089 [0.070, 0.121]	**< 0.001**
16	FM_{SM}	0.676 [0.639, 0.720]	0.559 [0.541, 0.575]	0.117 [0.090, 0.149]	**< 0.001**
32	FM_{SM}	0.725 [0.678, 0.771]	0.567 [0.553, 0.579]	0.158 [0.107, 0.208]	**< 0.001**
64	FM_{SM}	0.745 [0.703, 0.798]	0.648 [0.617, 0.683]	0.098 [0.072, 0.122]	**< 0.001**
128	FM_{SM}	0.758 [0.709, 0.816]	0.71 [0.658, 0.777]	0.048 [0.035, 0.060]	**< 0.001**
256	FM_{SM}	0.766 [0.712, 0.828]	0.746 [0.684, 0.815]	0.02 [0.006, 0.034]	**0.002**
512	FM_{SM}	0.768 [0.710, 0.833]	0.766 [0.698, 0.835]	0.003 [-0.007, 0.015]	0.582
1024	FM_{SM}	0.768 [0.708, 0.835]	0.775 [0.706, 0.842]	-0.007 [-0.020, 0.008]	0.375
2	FM_{SM}^{+MIMIC}	0.612 [0.579, 0.650]	0.543 [0.524, 0.562]	0.069 [0.048, 0.092]	**< 0.001**
4	FM_{SM}^{+MIMIC}	0.658 [0.608, 0.700]	0.529 [0.510, 0.550]	0.128 [0.090, 0.162]	**< 0.001**
8	FM_{SM}^{+MIMIC}	0.704 [0.647, 0.761]	0.561 [0.538, 0.583]	0.143 [0.105, 0.181]	**< 0.001**
16	FM_{SM}^{+MIMIC}	0.739 [0.678, 0.798]	0.559 [0.541, 0.575]	0.18 [0.128, 0.228]	**< 0.001**
32	FM_{SM}^{+MIMIC}	0.765 [0.702, 0.820]	0.567 [0.553, 0.579]	0.198 [0.133, 0.258]	**< 0.001**

64	FM_{SM}^{+MIMIC}	0.785 [0.725, 0.842]	0.648 [0.617, 0.683]	0.136 [0.099, 0.169]	**< 0.001**
128	FM_{SM}^{+MIMIC}	0.798 [0.74, 0.858]	0.71 [0.658, 0.775]	0.087 [0.071, 0.104]	**< 0.001**
256	FM_{SM}^{+MIMIC}	0.801 [0.743, 0.863]	0.746 [0.684, 0.817]	0.055 [0.041, 0.069]	**< 0.001**
512	FM_{SM}^{+MIMIC}	0.803 [0.745, 0.866]	0.766 [0.698, 0.835]	0.038 [0.026, 0.050]	**< 0.001**
1024	FM_{SM}^{+MIMIC}	0.807 [0.749, 0.869]	0.775 [0.706, 0.842]	0.033 [0.022, 0.046]	**< 0.001**

* Table shows mean AUROC (95% hierarchical bootstrap CI) by number of training samples
** bolded values indicate P < 0.05
Abbreviations: AUROC: area under the receiver operating characteristics curve. GBM: gradient boosting machines; FM_{SM}: external foundation model Stanford Medicine; FM_{SM}^{+}: external FM Stanford Medicine with continued pretraining - SK or MIMIC; SK: SickKids; MIMIC: Medical Information Mart for Intensive Care; CI – confidence interval.

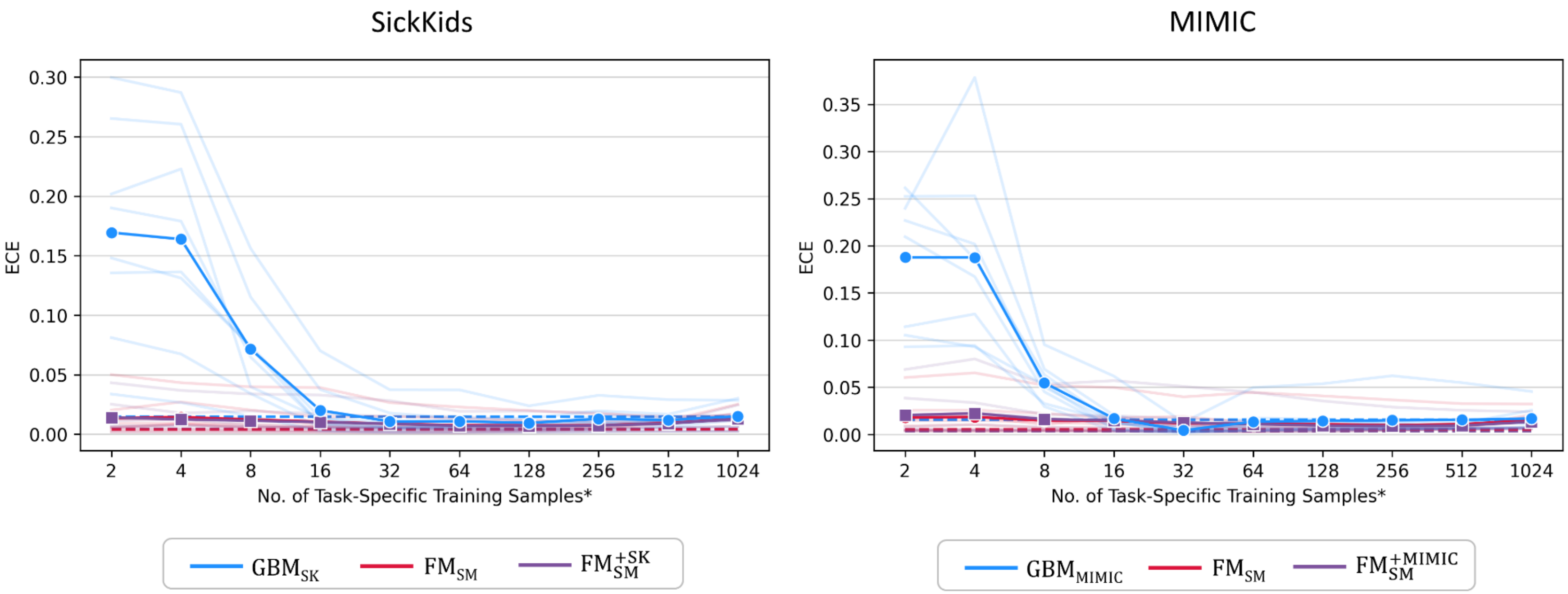

Supplementary Figure 2. Calibration of external foundation model (FM_{SM}), external foundation model with continued pretraining (FM^{+}_{SM}) and baseline GBM using decreasing training samples at SickKids and MIMIC. Bolded and faint lines indicate average and task-specific performance, respectively. Dashed lines indicate mean ECE of models trained on all training samples.

* The number of training examples for each class is up to half of the number of task-specific training samples.

Abbreviations: ECE: estimated calibration error; FM_{SM}: external foundation model Stanford Medicine; FM^{+}_{SM}: external foundation model Stanford Medicine with continued pretraining – SK or MIMIC; SK: SickKids; MIMIC: Medical Information Mart for Intensive Care; GBM: gradient boosting machines.

Supplementary Table 4. Comparing calibration of foundation models vs. baseline GBM using decreasing task-specific training samples*

Training Samples	Foundation Model	Foundation Model Performance	GBM Performance (Baseline)	Difference [Foundation Model – GBM]	P-value **
Dataset: SickKids					
2	FM_{SM}	0.014 [0.006, 0.026]	0.169 [0.112, 0.225]	-0.155 [-0.202, -0.103]	**< 0.001**
4	FM_{SM}	0.015 [0.007, 0.026]	0.162 [0.107, 0.223]	-0.148 [-0.198, -0.099]	**< 0.001**
8	FM_{SM}	0.013 [0.006, 0.022]	0.071 [0.045, 0.102]	-0.058 [-0.081, -0.038]	**< 0.001**
16	FM_{SM}	0.011 [0.005, 0.020]	0.020 [0.008, 0.036]	-0.009 [-0.016, -0.003]	**< 0.001**
32	FM_{SM}	0.010 [0.005, 0.016]	0.011 [0.005, 0.020]	-0.001 [-0.004, 0.000]	0.102
64	FM_{SM}	0.008 [0.004, 0.014]	0.011 [0.006, 0.020]	-0.003 [-0.007, -0.001]	**< 0.001**
128	FM_{SM}	0.008 [0.004, 0.013]	0.010 [0.006, 0.015]	-0.002 [-0.003, -0.001]	**< 0.001**
256	FM_{SM}	0.008 [0.005, 0.012]	0.013 [0.007, 0.020]	-0.005 [-0.008, -0.002]	**< 0.001**
512	FM_{SM}	0.010 [0.007, 0.013]	0.012 [0.007, 0.018]	-0.002 [-0.005, 0.000]	0.1
1024	FM_{SM}	0.014 [0.009, 0.018]	0.015 [0.009, 0.022]	-0.001 [-0.005, 0.001]	0.24
2	FM_{SM}^{+SK}	0.015 [0.007, 0.025]	0.169 [0.112, 0.225]	-0.154 [-0.202, -0.102]	**< 0.001**
4	FM_{SM}^{+SK}	0.013 [0.007, 0.022]	0.162 [0.107, 0.223]	-0.149 [-0.201, -0.100]	**< 0.001**
8	FM_{SM}^{+SK}	0.012 [0.006, 0.020]	0.071 [0.045, 0.102]	-0.059 [-0.083, -0.038]	**< 0.001**
16	FM_{SM}^{+SK}	0.011 [0.005, 0.019]	0.020 [0.008, 0.036]	-0.009 [-0.018, -0.002]	**< 0.001**
32	FM_{SM}^{+SK}	0.009 [0.004, 0.016]	0.011 [0.005, 0.020]	-0.002 [-0.004, -0.000]	**0.008**
64	FM_{SM}^{+SK}	0.008 [0.004, 0.013]	0.011 [0.006, 0.020]	-0.004 [-0.008, -0.001]	**0.004**
128	FM_{SM}^{+SK}	0.007 [0.004, 0.012]	0.01 0[0.006, 0.015]	-0.003 [-0.004, -0.001]	**< 0.001**
256	FM_{SM}^{+SK}	0.008 [0.005, 0.012]	0.013 [0.007, 0.020]	-0.005 [-0.009, -0.002]	**< 0.001**
512	FM_{SM}^{+SK}	0.010 [0.007, 0.012]	0.012 [0.007, 0.018]	-0.003 [-0.006, 0.001]	0.132
1024	FM_{SM}^{+SK}	0.013 [0.009, 0.018]	0.015 [0.009, 0.022]	-0.002 [-0.006, 0.001]	0.204
Dataset: MIMIC					
2	FM_{SM}	0.018 [0.008, 0.033]	0.188 [0.138, 0.232]	-0.169 [-0.209, -0.128]	**< 0.001**
4	FM_{SM}	0.018 [0.009, 0.034]	0.184 [0.135, 0.254]	-0.165 [-0.22, -0.125]	**< 0.001**
8	FM_{SM}	0.015 [0.007, 0.028]	0.055 [0.042, 0.070]	-0.040 [-0.045, -0.034]	**< 0.001**
16	FM_{SM}	0.015 [0.007, 0.027]	0.017 [0.008, 0.032]	-0.002 [-0.006, 0.002]	0.404
32	FM_{SM}	0.012 [0.006, 0.022]	0.004 [0.003, 0.007]	0.008 [0.003, 0.015]	**< 0.001**
64	FM_{SM}	0.012 [0.006, 0.023]	0.014 [0.007, 0.026]	-0.002 [-0.004, -0.001]	**< 0.001**
128	FM_{SM}	0.011 [0.006, 0.021]	0.015 [0.007, 0.028]	-0.003 [-0.007, -0.001]	**< 0.001**
256	FM_{SM}	0.010 [0.006, 0.019]	0.015 [0.007, 0.030]	-0.005 [-0.011, -0.001]	**< 0.001**
512	FM_{SM}	0.011 [0.007, 0.019]	0.015 [0.008, 0.028]	-0.004 [-0.010, -0.001]	**< 0.001**
1024	FM_{SM}	0.015 [0.010, 0.022]	0.017 [0.009, 0.027]	-0.002 [-0.006, 0.002]	0.426
2	FM_{SM}^{+MIMIC}	0.020 [0.009, 0.038]	0.188 [0.138, 0.232]	-0.166 [-0.206, -0.127]	**< 0.001**
4	FM_{SM}^{+MIMIC}	0.021 [0.010, 0.040]	0.184 [0.135, 0.254]	-0.162 [-0.214, -0.124]	**< 0.001**
8	FM_{SM}^{+MIMIC}	0.017 [0.008, 0.030]	0.055 [0.042, 0.070]	-0.038 [-0.044, -0.032]	**< 0.001**
16	FM_{SM}^{+MIMIC}	0.015 [0.006, 0.030]	0.017 [0.008, 0.032]	-0.001 [-0.004, 0.001]	0.214
32	FM_{SM}^{+MIMIC}	0.014 [0.006, 0.025]	0.004 [0.003, 0.007]	0.009 [0.003, 0.018]	**< 0.001**

64	FM_{SM}^{+MIMIC}	0.011 [0.005, 0.023]	0.014 [0.007, 0.026]	-0.003 [-0.004, -0.001]	**< 0.001**
128	FM_{SM}^{+MIMIC}	0.010 [0.005, 0.019]	0.015 [0.007, 0.028]	-0.005 [-0.009, -0.002]	**< 0.001**
256	FM_{SM}^{+MIMIC}	0.009 [0.005, 0.017]	0.015 [0.007, 0.030]	-0.006 [-0.014, -0.001]	**< 0.001**
512	FM_{SM}^{+MIMIC}	0.010 [0.007, 0.016]	0.015 [0.008, 0.028]	-0.005 [-0.013, -0.001]	**< 0.001**
1024	FM_{SM}^{+MIMIC}	0.014 [0.009, 0.018]	0.017 [0.009, 0.027]	-0.003 [-0.009, 0.001]	0.193

* Table shows mean ECE (95% hierarchical bootstrap CI) by number of training samples
** bolded values indicate P < 0.05
Abbreviations: ECE: expected calibration error. GBM: gradient boosting machines; FM_{SM}: external foundation model Stanford Medicine; FM_{SM}^{+}: external foundation model Stanford Medicine with continued pretraining - SK or MIMIC; SK: SickKids; MIMIC: Medical Information Mart for Intensive Care; CI – confidence interval

Supplementary Table 5. Comparing discrimination of external vs. local foundation models approaches at each pretraining sample size*

Proportion Pretraining Samples	External Foundation Model	External Foundation Model Performance	Local Foundation Model Performance	Difference [External – Local]	P-value
SickKids					
0.001	FM_{SM}	0.88 [0.826, 0.928]	0.794 [0.741, 0.843]	-0.086 [-0.112, -0.062]	**< 0.001**
0.01	FM_{SM}	0.88 [0.826, 0.928]	0.837 [0.788, 0.887]	-0.043 [-0.071, -0.02]	**< 0.001**
0.05	FM_{SM}	0.88 [0.826, 0.928]	0.866 [0.821, 0.91]	-0.014 [-0.033, 0.005]	0.142
0.1	FM_{SM}	0.88 [0.826, 0.928]	0.875 [0.831, 0.914]	-0.006 [-0.021, 0.011]	0.48
0.2	FM_{SM}	0.88 [0.826, 0.928]	0.891 [0.84, 0.937]	0.011 [-0.001, 0.023]	0.078
0.4	FM_{SM}	0.88 [0.826, 0.928]	0.891 [0.84, 0.936]	0.011 [-0.01, 0.025]	0.23
0.8	FM_{SM}	0.88 [0.826, 0.928]	0.9 [0.851, 0.941]	0.019 [0.006, 0.034]	**0.002**
0.001	FM_{SM}^{+SK}	0.879 [0.828, 0.922]	0.794 [0.741, 0.843]	-0.085 [-0.111, -0.061]	**< 0.001**
0.01	FM_{SM}^{+SK}	0.876 [0.824, 0.922]	0.837 [0.787, 0.887]	-0.039 [-0.065, -0.016]	**< 0.001**
0.05	FM_{SM}^{+SK}	0.884 [0.828, 0.933]	0.866 [0.821, 0.91]	-0.017 [-0.035, 0.002]	0.078
0.1	FM_{SM}^{+SK}	0.887 [0.833, 0.936]	0.875 [0.831, 0.914]	-0.013 [-0.03, 0.005]	0.166
0.2	FM_{SM}^{+SK}	0.891 [0.836, 0.938]	0.891 [0.84, 0.937]	-0.0 [-0.011, 0.013]	0.964
0.4	FM_{SM}^{+SK}	0.898 [0.845, 0.944]	0.891 [0.84, 0.936]	-0.006 [-0.028, 0.005]	0.366
0.8	FM_{SM}^{+SK}	0.899 [0.85, 0.941]	0.9 [0.851, 0.941]	0.0 [-0.014, 0.016]	0.956
MIMIC					
0.001	FM_{SM}	0.828 [0.775, 0.875]	0.762 [0.705, 0.812]	-0.066 [-0.089, -0.048]	**< 0.001**
0.01	FM_{SM}	0.828 [0.775, 0.875]	0.815 [0.775, 0.858]	-0.013 [-0.03, 0.013]	0.253
0.05	FM_{SM}	0.828 [0.775, 0.875]	0.833 [0.787, 0.88]	0.006 [-0.008, 0.023]	0.352
0.1	FM_{SM}	0.828 [0.775, 0.875]	0.836 [0.783, 0.884]	0.01 [-0.006, 0.024]	0.218
0.2	FM_{SM}	0.828 [0.775, 0.875]	0.84 [0.785, 0.889]	0.013 [-0.002, 0.025]	0.073
0.4	FM_{SM}	0.828 [0.775, 0.875]	0.845 [0.794, 0.892]	0.017 [0.003, 0.031]	**0.028**
0.8	FM_{SM}	0.828 [0.775, 0.875]	0.845 [0.774, 0.898]	0.018 [-0.01, 0.036]	0.183
0.001	FM_{SM}^{+MIMIC}	0.828 [0.765, 0.88]	0.761 [0.705, 0.812]	-0.066 [-0.088, -0.045]	**< 0.001**
0.01	FM_{SM}^{+MIMIC}	0.83 [0.766, 0.882]	0.815 [0.775, 0.858]	-0.015 [-0.036, 0.021]	0.39
0.05	FM_{SM}^{+MIMIC}	0.837 [0.768, 0.887]	0.833 [0.787, 0.88]	-0.003 [-0.021, 0.031]	0.849
0.1	FM_{SM}^{+MIMIC}	0.838 [0.77, 0.889]	0.836 [0.783, 0.884]	-0.0 [-0.017, 0.024]	0.991
0.2	FM_{SM}^{+MIMIC}	0.839 [0.769, 0.89]	0.84 [0.785, 0.889]	0.002 [-0.01, 0.02]	0.759
0.4	FM_{SM}^{+MIMIC}	0.844 [0.775, 0.894]	0.845 [0.794, 0.892]	0.001 [-0.012, 0.026]	0.925
0.8	FM_{SM}^{+MIMIC}	0.848 [0.782, 0.896]	0.845 [0.774, 0.898]	-0.002 [-0.021, 0.011]	0.804

* Table shows mean AUROC (95% hierarchical bootstrap CI) by proportion of pretraining cohort size

** Bolded values indicate P<0.05.

Abbreviations: AUROC: area under the receiver operating characteristics curve. FM_{SM}: external foundation model Stanford Medicine; FM_{SM}^{+}: external foundation model Stanford Medicine with continued pretraining - SK or MIMIC; SK: SickKids; MIMIC: Medical Information Mart for Intensive Care; CI: confidence interval.

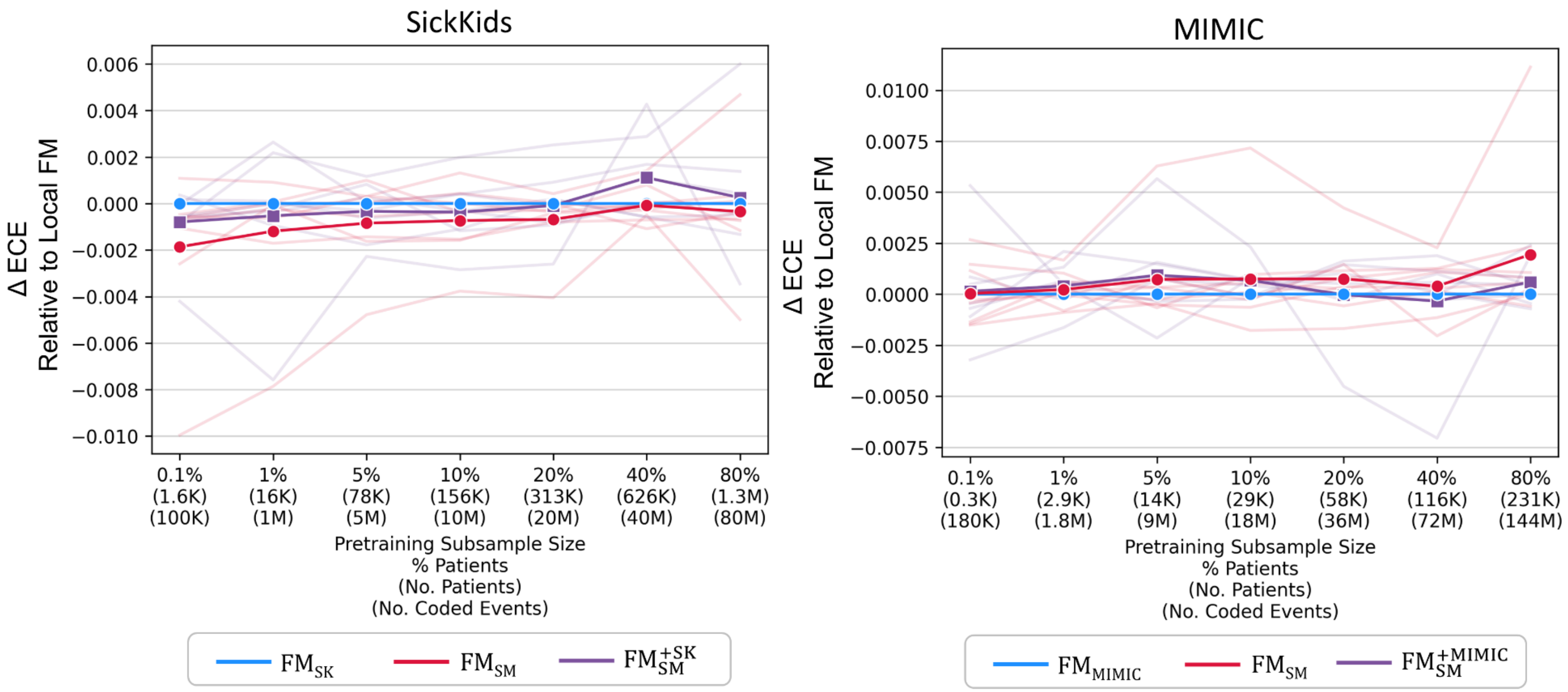

Supplementary Figure 3. Calibration of external foundation model (FM_{SM}) and external foundation model with continued pretraining (FM_{SM}^{+}) relative to local foundation model (FM_{SK} and FM_{MIMIC}) using decreasing pretraining sample size.

Bolded and faint lines indicate average and task-specific performance relative to local foundation models, respectively. The subsample size is not relevant for FM_{SM}, which did not undergo additional pretraining. Note, the model ECE scores are relative to the baseline local models (FM_{SK} and FM_{MIMIC}) and the absolute ECE does change across sample sizes.

Abbreviations: ECE: expected calibration error; FM_{SM}: external foundation model Stanford Medicine; FM_{SM}^{+}: external foundation model Stanford Medicine with continued pretraining – SK or MIMIC; FM_{SK}/FM_{MIMIC}: local foundation model – SK or MIMIC; SK: SickKids; MIMIC: Medical Information Mart for Intensive Care.

Supplementary Table 6. Comparing calibration of external vs. local foundation models at each pretraining sample size*

Proportion Pretraining Samples	External Foundation Model	External Foundation Model Performance	Local Foundation Model Performance	Difference [External – Local]	P-value
SickKids					
0.001	FM_{SM}	0.005 [0.003, 0.009]	0.007 [0.004, 0.011]	0.002 [-0.001, 0.004]	0.162
0.01	FM_{SM}	0.005 [0.003, 0.009]	0.007 [0.004, 0.01]	0.001 [-0.001, 0.003]	0.2
0.05	FM_{SM}	0.005 [0.003, 0.009]	0.006 [0.003, 0.01]	0.001 [-0.001, 0.002]	0.414
0.1	FM_{SM}	0.005 [0.003, 0.009]	0.006 [0.003, 0.009]	0.0 [-0.001, 0.002]	0.602
0.2	FM_{SM}	0.005 [0.003, 0.009]	0.006 [0.003, 0.009]	0.0 [-0.002, 0.002]	0.788
0.4	FM_{SM}	0.005 [0.003, 0.009]	0.005 [0.003, 0.009]	-0.0 [-0.002, 0.001]	0.924
0.8	FM_{SM}	0.005 [0.003, 0.009]	0.005 [0.003, 0.009]	0.0 [-0.002, 0.002]	0.94
0.001	FM_{SM}^{+SK}	0.006 [0.003, 0.009]	0.007 [0.004, 0.011]	0.001 [-0.001, 0.003]	0.226
0.01	FM_{SM}^{+SK}	0.006 [0.003, 0.009]	0.007 [0.004, 0.01]	0.001 [-0.001, 0.003]	0.518
0.05	FM_{SM}^{+SK}	0.006 [0.003, 0.01]	0.006 [0.003, 0.01]	0.0 [-0.001, 0.002]	0.64
0.1	FM_{SM}^{+SK}	0.005 [0.003, 0.009]	0.006 [0.003, 0.009]	0.0 [-0.001, 0.002]	0.594
0.2	FM_{SM}^{+SK}	0.006 [0.003, 0.01]	0.006 [0.003, 0.009]	0.0 [-0.002, 0.002]	0.994
0.4	FM_{SM}^{+SK}	0.006 [0.003, 0.01]	0.005 [0.003, 0.009]	-0.001 [-0.002, 0.001]	0.374
0.8	FM_{SM}^{+SK}	0.006 [0.003, 0.01]	0.005 [0.003, 0.009]	-0.0 [-0.002, 0.001]	0.924
MIMIC					
0.001	FM_{SM}	0.007 [0.004, 0.012]	0.007 [0.004, 0.012]	0.0 [-0.002, 0.002]	0.737
0.01	FM_{SM}	0.007 [0.004, 0.012]	0.007 [0.004, 0.012]	-0.0 [-0.002, 0.002]	0.992
0.05	FM_{SM}	0.007 [0.004, 0.012]	0.006 [0.003, 0.01]	-0.001 [-0.003, 0.001]	0.414
0.1	FM_{SM}	0.007 [0.004, 0.012]	0.006 [0.004, 0.01]	-0.001 [-0.003, 0.001]	0.516
0.2	FM_{SM}	0.007 [0.004, 0.012]	0.006 [0.003, 0.01]	-0.001 [-0.003, 0.001]	0.432
0.4	FM_{SM}	0.007 [0.004, 0.012]	0.006 [0.003, 0.011]	-0.001 [-0.003, 0.001]	0.524
0.8	FM_{SM}	0.007 [0.004, 0.012]	0.005 [0.003, 0.009]	-0.001 [-0.004, 0.0]	0.093
0.001	FM_{SM}^{+MIMIC}	0.007 [0.003, 0.012]	0.007 [0.004, 0.012]	0.0 [-0.002, 0.002]	0.876
0.01	FM_{SM}^{+MIMIC}	0.007 [0.004, 0.012]	0.007 [0.004, 0.012]	0.0 [-0.002, 0.002]	0.998
0.05	FM_{SM}^{+MIMIC}	0.007 [0.004, 0.011]	0.006 [0.003, 0.01]	-0.0 [-0.002, 0.001]	0.517
0.1	FM_{SM}^{+MIMIC}	0.006 [0.004, 0.011]	0.006 [0.004, 0.01]	-0.0 [-0.002, 0.001]	0.619
0.2	FM_{SM}^{+MIMIC}	0.006 [0.004, 0.009]	0.006 [0.003, 0.01]	0.0 [-0.001, 0.002]	0.961
0.4	FM_{SM}^{+MIMIC}	0.006 [0.004, 0.01]	0.006 [0.003, 0.011]	0.0 [-0.002, 0.002]	0.939
0.8	FM_{SM}^{+MIMIC}	0.006 [0.003, 0.01]	0.005 [0.003, 0.009]	-0.0 [-0.002, 0.001]	0.542

* Table shows mean ECE (95% hierarchical bootstrap CI) by proportion of pretraining cohort size

** Bolded values indicate P<0.05.

Abbreviations: ECE: expected calibration error. FM_{SM}: external foundation model Stanford Medicine; FM_{SM}^{+}: external foundation model Stanford Medicine with continued pretraining - SK or MIMIC; SK: SickKids; MIMIC: Medical Information Mart for Intensive Care; CI: confidence interval.

Supplementary Table 7. Comparing discrimination of local foundation models pretrained on all data with continued pretraining of FM_{SM} using increasing pretraining sample size*

Proportion Pretraining Samples	FM^{+}_{SM}	Local FM (FM_{SK} or FM_{MIMIC})	Difference [External – Local]	P-value
SickKids				
0.001	0.879 [0.828, 0.922]	0.900 [0.849, 0.942]	-0.020 [-0.033, -0.008]	**0.004**
0.01	0.876 [0.824, 0.922]	0.900 [0.849, 0.942]	-0.023 [-0.038, -0.010]	**< 0.001**
0.05	0.884 [0.828, 0.933]	0.900 [0.849, 0.942]	-0.016 [-0.032, -0.001]	**0.038**
0.1	0.887 [0.833, 0.936]	0.900 [0.849, 0.942]	-0.012 [-0.030, 0.004]	0.138
0.2	0.891 [0.836, 0.938]	0.900 [0.849, 0.942]	-0.008 [-0.027, 0.007]	0.306
0.4	0.898 [0.845, 0.944]	0.900 [0.849, 0.942]	-0.001 [-0.016, 0.013]	0.850
0.8	0.899 [0.850, 0.941]	0.900 [0.849, 0.942]	-0.001 [-0.017, 0.017]	0.932
MIMIC				
0.001	0.828 [0.765, 0.880]	0.850 [0.792, 0.898]	-0.022 [-0.038, -0.010]	**<0.001**
0.01	0.830 [0.766, 0.881]	0.850 [0.793, 0.898]	-0.020 [-0.037, -0.010]	**<0.001**
0.05	0.837 [0.768, 0.887]	0.850 [0.792, 0.898]	-0.014 [-0.032, -0.001]	**0.037**
0.1	0.838 [0.770, 0.889]	0.850 [0.792, 0.898]	-0.013 [-0.030, -0.001]	**0.042**
0.2	0.839 [0.769, 0.890]	0.850 [0.792, 0.898]	-0.011 [-0.028, -0.001]	**0.046**
0.4	0.844 [0.775, 0.894]	0.850 [0.792, 0.898]	-0.006 [-0.024, 0.005]	0.316
0.8	0.848 [0.782, 0.896]	0.850 [0.792, 0.898]	-0.003 [-0.016, 0.008]	0.575

* Table shows mean AUROC (95% hierarchical bootstrap CI) by proportion of pretraining cohort size

** Bolded values indicate P<0.05.

Abbreviations: AUROC: area under the receiver operating characteristics curve; FM^{+}_{SM}: external foundation model Stanford Medicine with continued pretraining - SK or MIMIC; FM_{SK}/FM_{MIMIC}: local foundation model – SK or MIMIC; SK: SickKids; MIMIC: Medical Information Mart for Intensive Care; CI: confidence interval.

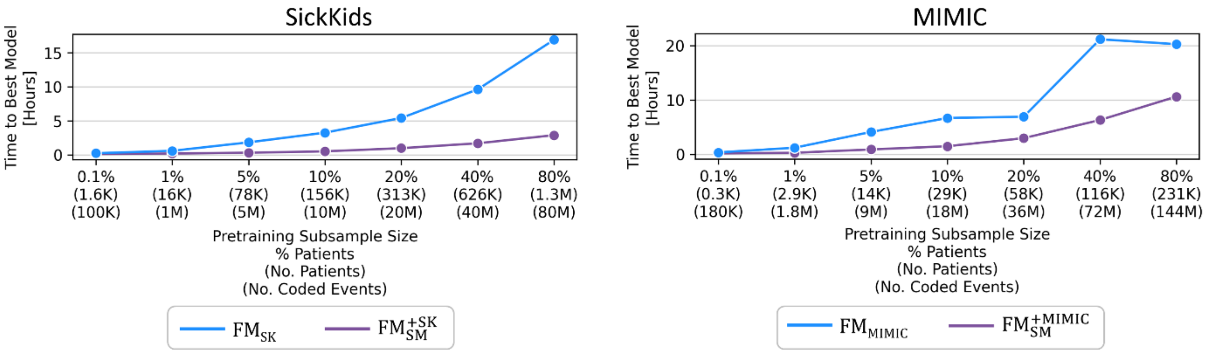

Supplementary Figure 4. Pretraining time for continued pretraining compared to local foundation models (FM_{SK} and FM_{MIMIC}) measured as the average time to best model across hyperparameter settings for each pretraining sample size.

Abbreviations: FM_{SM}^{+}: external foundation model Stanford Medicine with continued pretraining - SK or MIMIC; FM_{SK}/FM_{MIMIC}: local foundation model – SK or MIMIC; SK: SickKids; MIMIC: Medical Information Mart for Intensive Care.

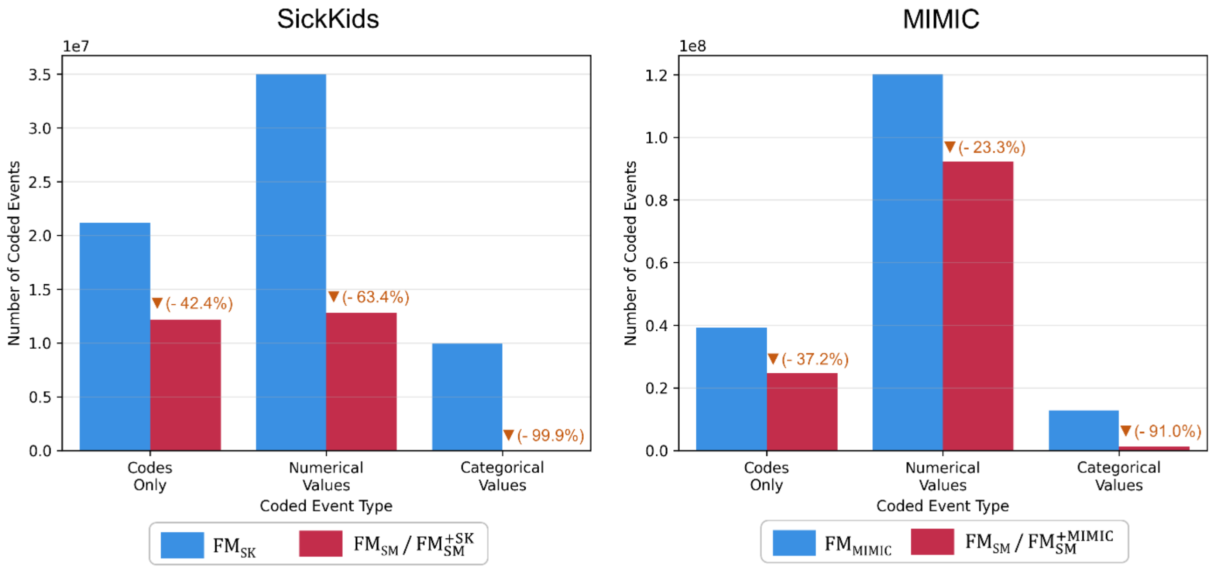

Supplementary Figure 5. Number of SickKids/MIMIC coded events across all patient timelines processed by local (blue) and external (red) foundation models, with values in orange indicating the relative percentage of coded events not processed (due to not being supported) by external foundation models. This involved an independent analysis in which we used each foundation model to process the entire timeline of each patient in the dataset.

Abbreviations: FM_{SM}: external foundation model Stanford Medicine; FM_{SM}^{+}: external foundation model Stanford Medicine with continued pretraining - SK or MIMIC; FM_{SK}/FM_{MIMIC}: local foundation model – SK or MIMIC; SK: SickKids; MIMIC: Medical Information Mart for Intensive Care.

Supplementary Table 8. Count-based featurization

This conventional featurization approach transforms each patient's electronic health records timeline into a count vector. Each patient's timeline is a sequence of clinical events $X = (x_1, x_2, \dots, x_n)$, where each x_i denoting the i-th code, encompassing any form of structured data obtained from the patient's EHR including diagnosis, lab test, or medication as examples. Each element in the resulting count vector represents the frequency of a specific clinical event (e.g. a diagnosis) occurring within a defined time window (e.g. 1 to 7 days) preceding the prediction time. Featurization was carried out over 3 time windows with respect to prediction time: 0-1 day, 1-7 days, and 7 days to the entire history. The count vectors for all patients were then combined into a high-dimensional (~30K for SK patients, and ~80K for MIMIC patients), sparse count matrix.

Supplementary Table 9. Hyperparameter settings for each model

Hyperparameter	Values
GBM	
lr	0.01, 0.1, 0.2
num_leaves	100, 300
boosting_type	“gbdt”, “dart”, “goss”
n_estimators	1000
L2-regularized logistic regression	
C	10^x where x ranges from -5 to 1 in 20 equal steps
max_iter	10,000
*Continued Pretraining and Pretraining of Local Foundation Models	
Learning rate	1e-4, 1e-5, 1e-6, 1e-7, 1-e8

*All foundation models utilized the same transformer architecture with 12 identical layers where each layer is consisted of 12 attention heads and 2 feedforward layers with a local attention mechanism.
